# Editorial: Molecular biology and treatment strategies for tumors of middle and inner ear

**DOI:** 10.3389/fneur.2025.1759916

**Published:** 2026-01-05

**Authors:** Oskar Rosiak, Wiesław Konopka, Andrew Fishman

**Affiliations:** 1 Department of Otolaryngology, Polish Mother's Memorial Hospital Research Institute, Lodz, Poland; 2 Department of Otolaryngology Head and Neck Surgery, University of Missouri, Columbia, MO, United States; 3 Department of Otolaryngology-Head and Neck Surgery, Medical University of Lublin, Lublin, Poland; 4 Department of Otorhinolaryngology (ORL)-Neurosurgery, BelMedic Clinical Center, Belgrade, Serbia; 5 Department of Otolaryngology, Audiology and Phoniatrics, Children's Hospital of Bydgoszcz, Bydgoszcz, Poland

**Keywords:** endolymphatic sac tumor, paraganglioma, sinonasal tumors, temporal bone tumors, vestibular schwannoma

## 1 Introduction

The six articles included in this Research Topic reflect developments in the understanding and management of tumors affecting the skull base, cerebellopontine angle, temporal bone and sinonasal cavity. These studies collectively highlight the importance of imaging standardization, individualized surgical planning, genetic risk stratification, and multidisciplinary care. In this Editorial, we summarize their contributions and discuss how they advance current paradigms in neuro-oncology and head-and-neck oncology.

## 2 Decision-making in vestibular schwannoma treatment

Vestibular schwannoma (previously also called acoustic neuroma) remains a central focus of skull base oncology being one of the most frequent cerebello-pontine angle tumors. In a narrative review, we provide “*Treatment planning for patients with acoustic neuroma*” (Fishman et al.) an updated synthesis of key patient-specific factors—including hearing status, vestibular symptoms, tumor size and location, and growth dynamics—that guide patient-specific treatment decisions. We underscore the balance between observation, microsurgical resection, and stereotactic radiosurgery, stressing the need to incorporate both short- and long-term functional hearing and balance outcomes into shared decision-making.

Complementing this, Zaczkowski et al. “*Pneumatization pattern of the temporal bone with volumetric assessment and its impact on the frequency of cerebrospinal fluid leak in patients undergoing vestibular schwannoma surgery via the retrosigmoid approach*” investigate the impact of temporal bone pneumatization on post-operative cerebrospinal fluid (CSF) leak risk following retrosigmoid vestibular schwannoma surgery. Their volumetric analysis demonstrates a significant association between increased pneumatization and higher CSF leak rates, suggesting a role for preoperative imaging-derived anatomical metrics in predicting complications and optimizing surgical planning.

## 3 Importance of standardization in diagnostic imaging headings

The complex anatomy of the nasal cavity and paranasal sinuses continues to pose diagnostic challenges. The expert consensus by Zhang et al. “*Chinese expert consensus on imaging examination and diagnosis of nasal cavity and paranasal sinus tumors*” offer a detailed, standardized imaging framework that integrates optimized CT and MRI protocols, radiation safety considerations, and recognition of tumor-specific imaging features. This guideline is particularly impactful for improving diagnostic accuracy, informing staging, and supporting multidisciplinary management of sinonasal tumors.

## 4 Rare temporal bone tumors

Two case-focused articles included in this Research topic highlight the diverse presentations and treatment challenges posed by rare temporal bone neoplasms.

Rosiak et al. “*Case report: Cochlear implantation for deafness caused by endolymphatic sac tumors in patients with von Hippel–Lindau syndrom*e” present a case of bilateral endolymphatic sac tumor (ELST) in a patient with von Hippel–Lindau syndrome, in which simultaneous cochlear implantation during tumor excision successfully restored hearing. This is an educational case of a rare cause of sudden deafness in an adolescent, which is subsequently treated by tumor removal and simultaneous cochlear implantation on one-side and complete tumor removal on contra-lateral side with hearing preservation. Their accompanying literature review emphasizes early audiological surveillance and demonstrates that cochlear implantation remains a viable strategy when profound hearing loss is present. Modern cochlear implants allow for magnetic resonance follow-up in oncologic patients.

Sun et al. “*Middle ear neuroendocrine tumor with multiple brain metastases: a case report and literature review*” report a rare case of a middle ear neuroendocrine tumor (MeNET) with multiple brain metastases, challenging prior assumptions regarding the low metastatic potential of this tumor. The authors identify several potential biomarkers of aggressive behavior—including Ki-67 and ATRX—that merit further study in larger patient cohorts.

## 5 Head and neck paraganglioma

A deeper understanding of the genetic background of head and neck neoplasms—and the identification of molecular and biochemical biomarkers—is urgently needed to move toward predictive and personalized medicine. In particular, unraveling not only the known susceptibility mutations, e.g., in the succinate dehydrogenase complex (SDH), but also additional genetic variants and pathway alterations may reveal novel therapeutic targets.

Radomska et al. “*Algorithm of genetic diagnosis for patients with head and neck paraganglioma—update*” provide a timely update to genetic diagnostic algorithms for head and neck paraganglioma (HNPGL), integrating data on subunits of the SDH complex (SDHD, SDHB, SDHC, SDHAF2) mutations and their associated clinical risk factors. Their work underlines the importance of genetic testing for guiding surveillance, identifying hereditary syndromes, and enabling early detection in at-risk relatives. The proposed diagnostic flowchart supports efficient, cost-effective testing in clinical practice.

This call for expanded genetic research is supported by accumulating evidence that HNPGLs are genetically heterogeneous: in addition to classic SDH-associated mutations, studies have identified potentially deleterious variants in non-susceptibility genes such as Aryl hydrocarbon receptor nuclear translocator (ARNT), Isocitrate dehydrogenase 2 (IDH2), p110α protein (PIK3CA), or telomerase reverse transcriptase (TERT) that may influence tumorigenesis and behavior (1). Efforts to integrate such broader molecular data with clinicopathologic features are likely to yield biomarkers useful for risk stratification, prognostication, and eventually targeted therapy.

## 6 Future directions

Together, the six contributions in this Research Topic highlight several converging themes that reflect current shifts in skull base and neuro-oncologic care. Foremost is the movement toward individualized, patient-centered surgical decision-making, informed by detailed anatomical evaluation and functional status rather than tumor size alone. Across multiple studies, the importance of high-quality, standardized imaging protocols emerges as a cornerstone for accurate diagnosis, staging, and treatment planning. At the same time, advances in molecular and genetic profiling increasingly support refined risk stratification and clinical surveillance strategies, particularly for tumor syndromes such as Von Hippel-Lindau. The authors underline the importance of functional preservation, not just radical resection alone, with hearing, balance, and neurological integrity recognized as essential outcome measures alongside tumor control. Finally, the rarity and biological heterogeneity of many skull base neoplasms underscore the necessity of multicenter collaboration, particularly to improve evidence generation for uncommon tumors such as endolymphatic sac tumors and middle ear neuroendocrine neoplasms.

Building on these insights, future research efforts should focus on integrating advanced imaging biomarkers with molecular and genetic signatures to establish predictive models for tumor behavior, where field for Artificial Intelligence emerges (2). The development of international registries will be essential to capture sufficient data for rare tumors including MeNET and ELST. Moreover, the incorporation of artificial intelligence–based imaging tools into clinical workflows represents a promising avenue for enhancing early detection, classification, and treatment monitoring of sinonasal and skull base tumors (3).

## 7 Conclusion

This Research Topic advances the field of skull base and neuro-oncologic tumor management through innovative approaches in imaging, surgical risk prediction, genetic diagnostics, and functional preservation. These contributions highlight the need for ongoing multidisciplinary collaboration and continued exploration of molecular and anatomical factors that influence outcomes.
